# MultiRTA: A simple yet reliable method for predicting peptide binding affinities for multiple class II MHC allotypes

**DOI:** 10.1186/1471-2105-11-482

**Published:** 2010-09-24

**Authors:** Andrew J Bordner, Hans D Mittelmann

**Affiliations:** 1 Mayo Clinic, 13400 East Shea Boulevard, Scottsdale, AZ 85259, USA; 2 School of Mathematical and Statistical Sciences, Arizona State University, P.O. Box 871804, Tempe, AZ 85287, USA

## Abstract

**Background:**

The binding of peptide fragments of antigens to class II MHC is a crucial step in initiating a helper T cell immune response. The identification of such peptide epitopes has potential applications in vaccine design and in better understanding autoimmune diseases and allergies. However, comprehensive experimental determination of peptide-MHC binding affinities is infeasible due to MHC diversity and the large number of possible peptide sequences. Computational methods trained on the limited experimental binding data can address this challenge. We present the MultiRTA method, an extension of our previous single-type RTA prediction method, which allows the prediction of peptide binding affinities for multiple MHC allotypes not used to train the model. Thus predictions can be made for many MHC allotypes for which experimental binding data is unavailable.

**Results:**

We fit MultiRTA models for both HLA-DR and HLA-DP using large experimental binding data sets. The performance in predicting binding affinities for novel MHC allotypes, not in the training set, was tested in two different ways. First, we performed leave-one-allele-out cross-validation, in which predictions are made for one allotype using a model fit to binding data for the remaining MHC allotypes. Comparison of the HLA-DR results with those of two other prediction methods applied to the same data sets showed that MultiRTA achieved performance comparable to NetMHCIIpan and better than the earlier TEPITOPE method. We also directly tested model transferability by making leave-one-allele-out predictions for additional experimentally characterized sets of overlapping peptide epitopes binding to multiple MHC allotypes. In addition, we determined the applicability of prediction methods like MultiRTA to other MHC allotypes by examining the degree of MHC variation accounted for in the training set. An examination of predictions for the promiscuous binding CLIP peptide revealed variations in binding affinity among alleles as well as potentially distinct binding registers for HLA-DR and HLA-DP. Finally, we analyzed the optimal MultiRTA parameters to discover the most important peptide residues for promiscuous and allele-specific binding to HLA-DR and HLA-DP allotypes.

**Conclusions:**

The MultiRTA method yields competitive performance but with a significantly simpler and physically interpretable model compared with previous prediction methods. A MultiRTA prediction webserver is available at http://bordnerlab.org/MultiRTA.

## Background

Class II MHC proteins expressed on the surfaces of professional antigen presenting cells (APCs) bind peptide fragments of extracellular proteins and thereby present them to helper T cells, which in turn recognize the MHC-bound fragments of non-self proteins to initiate an immune response. The resulting helper T cell response depends on the context and can include activation of macrophages, B cells and cytotoxic T cells or an inflammatory response. Because of it is crucial for a effective immune response, understanding peptide binding to class II MHC is important for understanding and treating human diseases. Misregulation of antigen recognition by class II MHC so that self proteins cause an immune response is responsible for autoimmune diseases. Indeed, the occurrence of many common autoimmune diseases are linked to particular class II MHC alleles [1-8]. Class II MHC epitopes also show promise in immunotherapies aimed at the treatment of allergies [9-14]. Finally, promiscuous class II MHC peptide epitopes, which bind to diverse MHC allotypes, can be employed in vaccines that are efficacious for a large proportion of the population [15-18].

In spite of their medical importance, the peptide binding preferences of different class II MHC proteins have not been fully characterized by experiments. This is largely because class II MHC genes are highly polymorphic, with hundreds of different MHC alleles, each potentially having unique peptide binding specificities. Although peptide binding affinities have been experimentally measured for some common alleles, the large number of MHC allotypes combined with the huge space of possible peptide sequences prevents comprehensive measurement of all peptide-MHC binding affinities. Computational methods can address this challenge by providing fast predictions of peptide-MHC binding affinities that can be used to guide further experimental studies.

Unlike class I MHC, which binds short peptides (8-11 residues), class II MHC generally binds longer peptides (15-25 residues) in a binding cleft that is open at both ends and so allows the bound peptide N- and C-termini to extend beyond the binding site. Thus only a short segment of the peptide, defined by a 9-mer core, interacts significantly with the MHC protein. This makes the prediction of peptide binding to class II MHC considerably more difficult than for class I MHC since the binding register of the peptide, *i.e*. the position of the 9-mer core segment within the peptide, must be predicted in addition to the binding affinity of the core segment to the class II MHC protein. This difficulty is reflected in the generally worse performance of class II MHC binding prediction methods as compared with class I MHC methods.

Sequence-based prediction methods, like the MultiRTA method described in this study, use experimental peptide binding data in order to identify sequence patterns that correlate with binding affinities. Early sequence-based prediction methods fit the total peptide binding energy [19,20], binding motif [21], geometric average binding affinity [22], or sequence alignment profile [23] in a particular register to a linear combination of contributions from individual residues, and represented them as binding profile matrices. The scores for all possible peptide binding registers were calculated and either the maximum value or sum were used as the final peptide binding score. Later methods employed various machine learning and data fitting approaches to prediction including partial least squares (PLS) [24,25], Gibbs sampling [26], linear programming [27], Support Vector Machines (SVMs) [28-30], kernel methods [31], or a combination of data fitting techniques [32]. Recently, we introduced the **R**egularized **T**hermodynamic **A**verage, or RTA, prediction method [33]. This method shares the complementary advantages of the former class of profile-based methods, namely an easily interpretable model with parameters representing the contributions of specific peptide residues to binding, and the latter class of machine learning-based methods, namely high prediction performance. It achieves this through the use of two techniques: (1) thermodynamic averaging over all possible binding registers and (2) incorporating a regularization constraint that reduces model overfitting by selecting only a subset of initial parameters.

The prediction methods discussed above are applicable only to an MHC allotype for which ample experimental peptide binding data is available, thus limiting their scope. Two previous methods, TEPITOPE [34] and NetMHCIIpan [35], were designed to make predictions for multiple HLA-DR allotypes. The TEPITOPE method accounts for specific interactions between peptide side chains and MHC pockets in order to make peptide binding predictions for MHC allotypes not in the training set but with a different combination of common binding pockets [34]. The more recent NetMHCIIpan method [35] accounts for MHC variability at the residue level and employs the SMM-align method [32] to identify the peptide 9-mer core followed by a consensus prediction using an ensemble of diverse artificial neural networks (ANNs) trained on sequence properties. In this study we introduce the MultiRTA method, a generalization of the RTA method that is able to predict peptide binding affinities for MHC allotypes not included in the training set by accounting for allele-specific MHC variation. This method shares the same advantages as RTA. Although it is much simpler than NetMHCIIpan and so has physically interpretable parameters, it is able to achieve comparable prediction accuracy to NetMHCIIpan while exceeding the accuracy of the other profile-based prediction model, TEPITOPE.

We first define the MultiRTA model and discuss the definition of MHC residue group variants used to account for the effect of different MHC types on peptide binding affinity. Next, we discuss parameter optimization and give a method for generating initial solutions. MultiRTA models for both HLA-DR and HLA-DP are then fit and their performance evaluated by leave-one-allele-out cross-validation, in which predictions are made for one allotype using a model fit to experimental binding data for the remaining MHC allotypes in the data set. The prediction performance on novel MHC allotypes is also directly evaluated using additional experimental peptide binding data. Finally, MultiRTA model parameters are analyzed in order to infer the primary determinants of peptide binding specificity for HLA-DR and HLA-DP.

## Methods

### Experimental peptide-MHC binding affinity data sets

In order to compare prediction results with NetMHCIIpan, we used the experimental peptide binding affinity data sets for 14 different HLA-DR allotypes employed in the paper describing the method [35]. The binding data in those sets were obtained from a relatively recent version of the IEDB database so that the latest database version yielded insufficient new data to warrant compiling a new data set. Also, in order to test the prediction performance for novel data, we collected experimental peptide binding data for HLA-DR allotypes that are different from the 14 types included in the training data and that are also among the 430 allotypes completely covered by the MultiRTA model residue groups (discussed in the Results section). As discussed below, sufficient data was found only for DRB1*1301. All quantitative binding data obtained by either radioactivity or fluorescence competition binding assays were collected from the Immune Epitope Database (IEDB) [36]. Because the NetMHCIIpan method requires that the peptides are at least 15 residues long, only data for such peptides were included so that that method could be evaluated on the data. A table of the final data set containing binding affinities for 127 peptides binding to DRB1*1301 is provided as Additional file 1. The HLA-DP binding data was taken from Sidney *et al*. 2010 [37]. All data was used except for the single residue mutation peptides employed in the SAAS analysis. This comprised data for known epitopes as well as peptides spanning a set of *Phleum pratense *antigens.

### MHC residue variant groups

The peptide binding specificity of each MHC allotype is determined by polymorphic MHC residues in the binding cleft that can potentially interact with the core peptide side chains. HLA-DR polymorphic residues were defined to be any MHC residue that contacts one of the 9 peptide core residues in any X-ray structure of an HLA-DR peptide-MHC complex, in which contacting residues have non-hydrogen atom separation < 4 Å and corresponding residues in different MHC types were determined by a multiple sequence alignment. Because all 14 HLA-DR allotypes in the data set have the same α chain, all polymorphic residues occur in the β chain. Likewise, the polymorphic residues for HLA-DP are defined in same way and again all polymorphic residues in the five data set allotypes are in the β chain. Next, for each of the 9 peptide core positions, variants of polymorphic MHC residues contacting each core peptide residue, defined both by residue number and type, were then collected into groups such that (1) all residue variants in a group always co-occur in the MHC types used for training and (2) the groups are the largest such groups satisfying condition (1). The resulting residue groups for HLA-DR (HLA-DP) are given in **Table S1 **(**Table S3**) and the variant residue types for each MHC residue number are listed in **Table S2 **(**Table S4**), all of which are in Additional file 2. For example, one group of HLA-DR MHC residues contacting peptide residue P4 consists of MHC residues 11A, 13C, 26N, and 28I. This means that residues 11, 13, 26, and 28 contact peptide residue P4 in at least one HLA-DR peptide-MHC complex and that these residue variants always appear together in each of the 14 training set MHC types.

We next explain the motivation for this definition of residue groups. As will be seen in the next section, the MultiRTA model assumes that the total peptide-MHC binding energy is a sum of contributions from all contacting pairs of peptide and MHC residues. Furthermore, the contribution from each peptide-MHC residue pair depends on the particular peptide and MHC residue types. One could define a model in terms of individual MHC residues, rather than the variant groups, however it would have significantly more parameters to fit. Furthermore, the relative energy contributions for individual residues within the same residue group cannot be determined from the training data. Using the example above, the model without residue groups would have four separate parameters for P4 interacting with each of MHC residues 11A, 13C, 26N, and 28I. However, because these residues always co-occur in the training set MHC types there is no procedure for determining the relative values of parameters for P4 interacting with each of these four residues individually. Thus using residue groups provides the most concise description of the interaction energy without introducing spurious underdetermined parameters that would make the model more difficult to optimize and so may compromise its accuracy.

### MultiRTA model

As in the RTA model, the total binding affinity of peptide *k*, Δ*G*^(*k*)^, is calculated as a Boltzmann-weighted average over the binding affinities in different registers, $\Delta G_{M}^{\left(k\right)}$,

(1) $$\Delta G^{\left(k\right)}=\frac{\sum_{M=0}^{L\left(k\right)-9}\Delta G_{M}^{\left(k\right)}\exp\left(\Delta G_{M}^{\left(k\right)}/kT\right)}{\sum_{M=0}^{L\left(k\right)-9}\exp\left(\Delta G_{M}^{\left(k\right)}/kT\right)},$$

in which L(k) is the length of peptide k. Next, we define a variable *z_i,T(k),l_*, that is equal to 1 if the MHC type corresponding to peptide *k*, T(k), contains residue group l for peptide residue P*i *and is equal to 0 otherwise. Likewise the amino acid sequence of peptide *k *is also encoded by a binary array $x_{i,j}^{\left(k\right)}$ that is equal to 1 if the residue at position *i *is of type *j*, with residue types numbered from 1 to 20. The binding affinity of peptide *k *in register *M *is then

(2) $$\Delta G_{M}^{\left(k\right)}=\sum_{i=1}^{9}\sum_{j=1}^{20}\sum_{l=1}^{N_{g}\left(i\right)}\beta_{ijl}x_{i+M,j}^{\left(k\right)}z_{i,T\left(k\right),l}+\sum_{i=1}^{9}\sum_{j=1}^{20}\gamma_{ij}x_{i+M,j}^{\left(k\right)},$$

in which N_g_(*i*) is the number of residue groups for peptide core residue P*i*. Parameters β_ijl _are the contribution to the total binding affinity from the peptide core residue P*i *of type *j *contacting the MHC residues in group *l*. Parameters γ_ij _are the contribution of peptide core residue Pi of type j interacting with the invariant contacting MHC residues common to all MHC types in the training set. Note that the second term has the same form as the RTA model, which is only applicable to a single MHC type. In order to simplify **Eq. 2**, we define an additional residue group with index *l* = 0 for the invariant residues so that the γ_ij _parameters are absorbed into the β_ijl_. The corresponding variables *z_i,T(k),0 _*are then equal to 1 for all *i *and *k*. **Eq. 2 **then becomes

(3) $$\Delta G_{M}^{\left(k\right)}=\sum_{i=1}^{9}\sum_{j=1}^{20}\sum_{l=0}^{N_{g}\left(i\right)}\beta_{ijl}x_{i+M,j}^{\left(k\right)}z_{i,T\left(k\right),l}$$

As in the RTA model, an L^1 ^regularization constraint is included in order to reduce overfitting. The constraint has the form

(4) $$\sum_{i=1}^{9}\sum_{j=1}^{20}\sum_{l=0}^{N_{g}\left(i\right)}\left|\beta_{ijl}\right|\leq t.$$

This constraint is particularly important for MultiRTA since the model has a large number of parameters, 4650 for HLA-DP and 23220 for HLA-DR, relative to the quantity of training data. Even for the RTA model, which has only 180 parameters, the constraint was found to significantly improve the prediction performance on novel data, as assessed by cross-validation. The L^1 ^constraint, which is also used in lasso regression [38], has the desirable property that an increasing number of parameters become zero as the cutoff *t *is lowered. In effect, the constraint is performing model selection by only including the most relevant set of parameters. This is not the case with the commonly used L^2 ^constraint, such as that employed in ridge regression, in which the unimportant parameters are reduced in magnitude rather than set to zero as the constraint cutoff is lowered. As with the RTA model, the constraint in **Eq. 4 **was active for all of the optimal model solutions so that many parameters were zero.

The model parameters, β_ijl_, were then fit by minimizing the mean square error (MSE)

(5) $$\text{MSE}=\frac{1}{N}{\sum_{k=1}^{N}\left[\Delta G^{\left(k\right)}-\Delta G_{\text{exp}}^{\left(k\right)}\right]}^{2}$$

between the predicted binding affinities, Δ*G*^(*k*)^, and the experimental ones, $\Delta G_{\text{exp}}^{\left(k\right)}$, subject to the constraint in **Eq. 4**.

### Initial MultiRTA solution from combining RTA parameters for all MHC types

Finding optimal parameter values by minimizing the MSE in **Eq. 5 **subject to the constraint in **Eq. 4 **is challenging because of the large number of parameters. An initial solution for MultiRTA parameters can be obtained by combining optimal parameters from single MHC type RTA models. Starting the solver with this solution improves its convergence and speed.

Consider MultiRTA restricted to data from a single MHC type with index *a*. The expression for the energy of peptide *k *binding in register *M *is

(6) $$\Delta G_{M}^{\left(k\right)}=\sum_{i=1}^{9}\sum_{j=1}^{20}\left[\sum_{l=0}^{N_{g}\left(i\right)}\beta_{ijl}z_{ial}\right]x_{i+M,j}^{\left(k\right)}.$$

Likewise in the RTA model for the same MHC type with parameters (*β_a_*)*_ij_* the same quantity is

(7) $$\Delta G_{M}^{\left(k\right)}=\sum_{i=1}^{9}\sum_{j=1}^{20}{\left(\beta_{a}\right)}_{ij}x_{i+M,j}^{\left(k\right)}.$$

It may be seen that $\Delta G_{M}^{\left(k\right)}$ calculated in MultiRTA is obtained by replacing (*β_a_*)*_ij _*by the expression in square brackets in **Eq. 6**. Thus we seek an initial solution for the MultiRTA model, $\beta_{ijl}^{0}$, that minimizes sum of square differences, r^2^, between these two quantities over all MHC types

(8) $$r^{2}={\sum_{a=1}^{N_{T}}\left(\sum_{l=0}^{N_{g}\left(i\right)}\beta_{ijl}^{0}z_{ial}-{\left(\beta_{a}\right)}_{ij}\right)}^{2}.$$

For fixed *i*,*j*, we simplify the notation by defining the matrix **Z **by **Z***_al _*= Z*_ial_*, the vector **b **by $b_{l}=\beta_{ijl}^{0}$, and the vector **c **by **c***_a _*= (*β*_a_)*_ij _*so that the initial solution that minimizes the residual *r *in **Eq. 8**, is now expressed as *r *= |**Zb **- **c**|. This is readily solved by first calculating the singular value decomposition (SVD) of **Z**, **Z **= **UWV***^T^*, and then using it to calculate its approximate pseudoinverse, up to a tolerance parameter ε, from **Z**^+ ^= **VW**^+^**U***^T^*. The matrix **W**^+ ^is defined in terms of the diagonal matrix **W **as

(9) $$W_{ij}^{+}=\{\begin{array}{cc} 1/W_{ij} & W_{ij}\geq \varepsilon \\ 0 & W_{ij}<\varepsilon \end{array}.$$

The best fit solution for each choice of *i*,*j *indices is then simply **Z**^+^**c**.

### Parameter optimization

To solve the optimization problem minimizing **Eq. 5 **under the constraint in **Eq. 4 **one first splits the variable β_ijl _into the difference of two nonnegative variables $\beta_{ijl}^{+}$ and $\beta_{ijl}^{-}$, so that $\beta_{ijl}=\beta_{ijl}^{+}-\beta_{ijl}^{-}$. The constraint **Eq. 4 **then has as the left side the sum over both new variables. Overall one has a linearly constrained nonlinear and nonconvex optimization problem. In principle, global optimization methods would have to be applied to find the global minimizer. Due to the dimensions of the problems these would be non-deterministic methods such as some of the many metaheuristics, which include simulated annealing and genetic algorithms. These methods require a large number of evaluations and due to their stochastic character would have to be run several times in order to increase the likelihood of finding the global optimum, although a guarantee for that is impossible. After initial tests we decided to instead use local solvers. These are very efficient and through various measures the chances of getting very good local minima can be increased substantially.

Using local solvers with "multistart" or several often randomly generated starting guesses is another way of solving global optimization problems. It is also implemented in several software packages. We used random starting guesses, varied in suitable ranges, for both the parameters β_ijl _and the bound t in **Eq. 4**. It is significant that the best solutions were obtained with values of the regularization parameter t that were small enough to restrict the fit β_ijl _values. This shows that the additional L^1 ^constraint in **Eq. 4 **helps alleviate overfitting. In order to be able to easily call a variety of solvers we phrased the problem in the modeling language AMPL [39]. In order not to have to list many separate citations we state that we used the applicable (NLP) solvers, particularly IPOPT and SNOPT, installed at NEOS (Network Enabled Optimization Server, http://neos.mcs.anl.gov/) but run locally, not through this free service in which we (HDM) are also heavily involved.

In the way described above we generated the values in Table 2. For Table 1, additional advantage was taken of the initial values obtained as in the preceding section to speed up convergence.

**Table 1:** Comparison of HLA-DR prediction results for MultiRTA, NetMHCIIpan, and TEPITOPE using the same data sets

	MultiRTA			NetMHCIIpan		TEPITOPE	
				
MHC β chain allele	AUC	RMS error (kcal/mol)	Correlation coefficient	AUC	Correlation coefficient	AUC	Number of data
DRB1*0101	**0.801**	1.33	**0.619**	0.778	0.570	0.720	5166
DRB1*0301	**0.751**	1.36	0.438	0.746	**0.449**	0.664	1020
DRB1*0401	0.763	1.56	0.534	**0.775**	**0.598**	0.716	1024
DRB1*0404	0.835	1.33	0.623	**0.852**	**0.684**	0.770	663
DRB1*0405	**0.808**	1.28	0.566	**0.808**	**0.597**	0.759	630
DRB1*0701	0.817	1.51	0.620	**0.825**	**0.655**	0.761	853
DRB1*0802	0.786	1.45	0.523	**0.841**	**0.631**	0.766	420
DRB1*0901	**0.674**	2.01	0.380	0.653	**0.388**	NA	530
DRB1*1101	**0.819**	1.46	**0.603**	0.799	0.588	0.721	950
DRB1*1302	**0.698**	1.68	**0.365**	0.658	0.351	0.652	498
DRB1*1501	0.729	1.57	0.513	**0.738**	**0.535**	0.686	934
DRB3*0101	**0.813**	1.10	**0.603**	0.716	0.444	NA	549
DRB4*0101	**0.746**	1.61	**0.508**	0.724	0.469	NA	446
DRB5*0101	0.788	1.60	0.543	**0.831**	**0.633**	0.680	924

The results for MultiRTA and NetMHCIIpan were obtained using leave-one-allele-out cross-validation, in which predictions are made for one allotype using a model trained on the data for the remaining allotypes. The largest AUC and correlation coefficient values for each MHC allotype are highlighted in bold.

**Table 2:** HLA-DP leave-one-allele-out cross-validation results for MultiRTA

MHC β chain allele	AUC	RMS error (kcal/mol)	Correlation coefficient	Number of data
DPB1*0101	0.892	2.74	0.706	481
DPB1*0201	0.890	1.99	0.718	474
DPB1*0401	0.903	0.960	0.730	552
DPB1*0402	0.901	0.981	0.698	537
DPB1*0501	0.871	1.01	0.628	475

### Binding affinity variation at each peptide core residue

The variation in binding affinity due to different residues at each of the nine peptide core residues was calculated for each MHC type in the training set in order to estimate the importance of each core residue to peptide-MHC binding. So-called anchor residues are expected be important for binding specificity and so have large variation. The variation at each core position P*i *for MHC type *T *was calculated as the standard deviation in the binding affinity contribution from residue *j *at that position, Δ*G_T,i,j_*, defined by

(10) $$\Delta G_{T,i,j}\equiv \sum_{l=0}^{N_{g}\left(i\right)}\beta_{ijl}z_{i,T,l}$$

and the standard deviation was calculated in the usual way as

(11) $$\begin{array}{c} \sigma_{T,i}=\sqrt{\frac{1}{20}\sum_{j=1}^{20}{\left(\Delta G_{T,i,j}-\langle \Delta G_{T,i}\rangle \right)}^{2}}\\ \langle \Delta G_{T,i}\rangle =\frac{1}{20}\sum_{j=1}^{20}\Delta G_{T,i,j} \end{array}$$

## Results and Discussion

### Allotype coverage

The MHC variation near the peptide binding site is accounted for in the MultiRTA model through residue variant groups (described in the Methods section). Residue variant groups are defined so that they are the most efficient description of allele-specific variations in MHC residue contacting each peptide core residue. Accurate MultiRTA binding affinity predictions can be made for a novel MHC allotype, not included in the training data, if model parameters are defined for all of its residue variant groups, *i.e*. each residue variant group is present in at least one training set MHC allotype.

In order to determine model parameters, z_iTl_, for novel MHC allotypes we calculated the residue groups present in all HLA-DR and DP allotypes with available β chain sequences in the IMGT/HLA database [40,41]. A total of 430 out of 572 HLA-DR allotypes and 10 out of 36 HLA-DP allotypes had all residue variant groups accounted for in the respective MultiRTA models. The lower percentage of HLA-DP allotypes (28%) covered compared with HLA-DR ones (75%) can be explained by the smaller number of allotypes with known amino acid sequences, the smaller number of allotypes represented in the training set, and their lower diversity. A prediction method such as TEPITOPE accounts for MHC variation at the pocket level rather than the residue level. This coarser description of MHC variation has the effect of reducing the number of HLA-DR allotypes covered by the model to only 148, significantly lower than residue level models such as MultiRTA and NetMHCIIpan and so limits its applicability to different MHC allotypes.

HLA-DR prediction methods like MultiRTA and NetMHCIIpan that account for residue level MHC variation are only parameterized for the 430 allotypes with MHC variants accounted for in the training set and so, strictly speaking, can only reliably predict binding affinities for this subset of allotypes. In other words, no method can differentiate between peptide binding preferences for two MHC allotypes whose interacting residue differences are not accounted for in the training set. This limitation in allotype coverage is due to variation in peptide-interacting MHC residues among the allotypes included in the training set and so does not depend on the nature of the prediction algorithm. In spite of this limitation, such methods can be applied to any HLA-DR allotype but at the expense of lower prediction accuracy for allotypes with MHC residue variants not represented in the training set, *i.e*. outside of the set of 430 types. The accuracy is expected to decrease in proportion to the number of MHC residue variations for the MHC allotype of interest that are missing from the training set. In MultiRTA, parameters for missing residue variant groups can be simply set to zero. The lowest percentage of MHC variant residue groups covered by the MultiRTA model among all MHC types was 83% for HLA-DR and 58% for HLA-DP. Thus, while MultiRTA should yield accurate results for almost all HLA-DR allotypes, its accuracy is expected to be lower for some HLA-DP allotypes with large percentages of missing MHC residue variant groups. The coverage will only increase in the future as new peptide binding data becomes available for other allotypes.

### Cross-validation results

MultiRTA prediction performance for 14 different HLA-DR allotypes was evaluated using the data sets from the NetMHCIIpan paper [35] in order to compare it with that method as well as with TEPITOPE [34]. Leave-one-allele-out cross-validation, as was used for the NetMHCIIpan results, was also used for MultiRTA in order to estimate its prediction accuracy for novel MHC allotypes, not included in the training set. This procedure involved making predictions for each MHC allotype using a model fit using data for the remaining MHC allotypes. The prediction results for HLA-DR, shown in Table 1, indicate that MultiRTA achieves comparable performance to NetMHCIIpan and significantly better performance than TEPITOPE. The AUC statistics are highly correlated between MultiRTA and NetMHCIIpan (ρ = 0.82), suggesting that the variable performance between alleles is due to characteristics of the data sets themselves and not to differences between the prediction models. In Nielsen *et al*. 2008 [35], this variability in accuracy was found to be correlated with the similarity of the test MHC allele to the nearest training set allele, except for a few outliers that did not follow this trend.

Table 2 shows the leave-one-allele-out cross-validation results for HLA-DP. The discrimination between binders and non-binders, reflected by the AUC statistic, is better for HLA-DP than for HLA-DR. This can be explained by the higher degree of similarity between the peptide proximal residues in different HLA-DP MHC proteins, as compared with the HLA-DR. The higher prediction accuracy for HLA-DP is also reflected in the correlation coefficients. The RMS error shows more variable prediction performance for the different HLA-DP allotypes, with lower values than any obtained for HLA-DR allotypes for three HLA-DP allotypes but high RMS error values for DPB1*0101 and DPB1*0201. The high RMS error for DPB1*0101 and DPB1*0201 is due to predicted binding affinities that are systematically lower than experimental values, with average differences of 2.57 and 1.7 kcal/mol, respectively. In summary, except for a systematic downward shift in binding affinities for DPB1*0101 and DPB1*0201, the statistics indicate that the prediction performance for HLA-DP is even better than for HLA-DR.

We also examined the prediction performance using a single-allotype RTA model for the most similar allotype, as determined by overall MHC amino acid sequence similarity. The results for HLA-DR and HLA-DP are shown in Table 3 and Table 4, respectively. A comparison with the MultiRTA results for HLA-DR in Table 1 shows that the MultiRTA performs better, as measured by AUC (Wilcoxon signed rank test p-value = 1.8 × 10^-4^). This demonstrates that incorporating information on multiple allotypes improves the prediction accuracy over the nearest single-type model. Nielsen et al. 2008 [35] arrived at a similar conclusion for their NetMHCIIpan method. A comparison of the results for HLA-DP in Table 4 and Table 2 does not show a statistically significant difference in AUC values (p-value = 6.3 × 10^-2^). This is probably due to both the small number of HLA-DP allotypes represented and their higher similarity in peptide binding preferences.

**Table 3:** Predictions for each HLA-DR allotype using a single-type RTA model trained on data for the closest MHC allotype

Test MHC allotype	Closest training MHC allotype	AUC	RMS error (kcal/mol)	Correlation coefficient
DRB1*0101	DRB1*1501	0.646	1.86	0.306
DRB1*0301	DRB1*1302	0.628	1.89	0.170
DRB1*0401	DRB1*0405	0.656	1.93	0.348
DRB1*0404	DRB1*0401	0.745	1.46	0.483
DRB1*0405	DRB1*0401	0.732	1.43	0.403
DRB1*0701	DRB1*0901	0.681	1.72	0.382
DRB1*0802	DRB1*1101	0.793	1.58	0.505
DRB1*0901	DRB1*0701	0.671	1.81	0.388
DRB1*1101	DRB1*1302	0.628	1.89	0.264
DRB1*1302	DRB1*1101	0.646	1.85	0.302
DRB1*1501	DRB1*0101	0.670	2.11	0.363
DRB3*0101	DRB1*0301	0.654	1.59	0.322
DRB4*0101	DRB1*0101	0.665	2.05	0.295
DRB5*0101	DRB1*0101	0.736	2.05	0.433

**Table 4:** Predictions for each HLA-DP allotype using a single-type RTA model trained on data for the closest MHC allotype

Test MHC allotype	Closest training MHC allotype	AUC	RMS error (kcal/mol)	Correlation coefficient
DPB1*0101	DPB1*0501	0.883	1.13	0.660
DPB1*0201	DPB1*0402	0.863	1.13	0.669
DPB1*0401	DPB1*0402	0.872	1.12	0.633
DPB1*0402	DPB1*0201	0.824	1.20	0.623
DPB1*0501	DPB1*0101	0.876	1.10	0.661

### Prediction performance for novel allotypes

We further evaluated the performance of MultiRTA on novel allotypes by comparing predictions with experimental binding affinity data for 103 overlapping peptides derived from four different antigens (bee venom phospholipase A_2 _[42], human LAGE-1 [43], dog allergen Can f 1 [44], and HIV Nef [45]) binding to 7 different HLA-DR allotypes. Such sets of binding affinities for multiple overlapping peptides from each protein arguably provides unbiased data for reliable prediction performance estimates. As in the cross-validation described above, a leave-one-allele-out prediction was made using a MultiRTA model fit only to data for other MHC allotypes in order to estimate performance for novel allotypes. The peptide binding data was downloaded from the Dana-Farber Repository for Machine Learning in Immunology (DFRMLI) web site http://bio.dfci.harvard.edu/DFRMLI/. MultiRTA prediction AUC values for each allotype are shown in Table 5. AUC confidence intervals were also calculated in order to quantify the uncertainty in the AUC values due to the small number of binders present in some of the data sets. A comparison of the corresponding AUC values between the DFRMLI data results and the cross-validation results in Table 1 showed no significant difference in AUC values (Wilcoxon signed rank test p-value = 0.16). In addition, the cross-validation AUC values are within the confidence intervals for the DFRMLI values. Thus MultiRTA does not appear to significantly lose accuracy when applied to such overlapping peptide data sets, which are similar to those encountered in actual epitope prediction problems.

**Table 5:** MultiRTA leave-one-allele-out prediction results for the DFRMLI compilation of experimental binding data for overlapping peptides from four different antigens

MHC allotype	AUC (± 95% CI)	Number of binders
DRB1*0101	0.798 (± 0.096)	23
DRB1*0301	0.659 (± 0.177)	11
DRB1*0401	0.787 (± 0.104)	22
DRB1*0701	0.797 (± 0.092)	17
DRB1*1101	0.772 (± 0.100)	29
DRB1*1301	0.722 (± 0.361)	2
DRB1*1501	0.639 (± 0.143)	15

The data set for each allotype contains 103 peptides with measured binding affinities. The 95% confidence intervals for AUC were calculated from the standard deviation of the linearly related Somer's D_xy _statistic ($\sigma_{\text{AUC}}=\frac{1}{2}\sigma_{Dxy}$) using the Hmisc package in R. Data sets with fewer binders have corresponding higher uncertainty in their AUC values.

We also tested the generality of MultiRTA by comparing predictions with IEDB data for 127 peptides binding to HLA-DRB1*1301. Binding data sets were available for other MHC allotypes however there were either too few data per allotype (< 20) or too few binders (< 5) to obtain confident prediction statistics and so they were not considered. The overall AUC, RMSE, and correlation coefficients were 0.783, 1.65 kcal/mol, and 0.469 for MultiRTA and 0.722, 1.40 kcal/mol, and 0.411 for NetMHCIIpan. The higher AUC and correlation coefficient values for MultiRTA reflect the results in Table 1 for the closely related HLA-DRB1*1302 allotype (shown in Table 1). The only residue group difference between HLA-DRB1*1301 and HLA-DRB1*1302 is the G86/V86 MHC variation near peptide residue P1, discussed in the next section, so that only the binding preferences at P1 differ. The inclusion of HLA-DRB1*1302 binding data in the training sets for both MultiRTA and NetMHCIIpan explains the higher statistics obtained for HLA-DRB1*1301 than for the leave-one-allele-out results in Table 1, in which data for allotypes closely related to HLA-DRB1*1302 are absent from training data.

### Primary determinants of binding affinity as inferred from model parameters

As mentioned above, an important advantage of MultiRTA over methods that use sophisticated machine learning algorithms such ANNs is the interpretability of its parameters. Each parameter, β_ijl_, represents the contribution to the total binding affinity of peptide residue type *j *at position P*i *interacting with MHC residue group *l*. A list of these parameter values, in order of their magnitude, or importance to the overall binding affinity, are given as Additional files 3 and 4.

An analysis of the largest magnitude parameters at each peptide core position reveals peptide-MHC interactions that contribute to both promiscuous and allele-specific peptide binding. Promiscuous interactions involve the peptide residue interacting with the MHC residue conserved among all training alleles and represented by β_ijl _with *l* = 0. We begin by looking at the HLA-DR parameters. The largest parameters at position P1 are from conserved MHC interactions with peptide residues having hydrophobic or aromatic side chains, Phe, Ile, Leu, Val, Tyr, Met, or Trp. This preference can explained by examining peptide-MHC X-ray structures; the P1 side chain fits into a hydrophobic pocket in the MHC protein [46]. There are only two MHC variant residues contacting P1, either a Gly or Val at residue β86. The largest model parameters show a preference for the larger aromatic residues (Phe, Tyr, and Trp) with the G86 variant and the remaining smaller hydrophobic residues with the V86 variant. This effect of the G86/V86 dimorphism on peptide binding was previous described [47,48] and can be attributed to the larger pocket of the G86 variant accommodating the larger aromatic P1 side chains. The magnitudes of the largest parameters at P1 are larger than those at any of the other eight peptide core positions, indicating weaker interactions at P2-P9. The variability of different HLA-DR alleles near P1 is also lower than at most other positions since the G86/V86 dimorphism comprises all variation at this site while other sites, except P8, have significantly more variant residue groups. Both of these factors, weaker interactions and lower diversity of contacting MHC residues, likely contribute to the observed weaker sequence preferences at peptide residue positions P2-P9.

The peptide-MHC interaction parameters can also be compared with an experimental study of binding motifs determined from the selection of strong binders from an unbiased and diverse set of peptides expressed in a phage display library [49]. The study examined peptide binding preferences of the DRB1*0101, DRB1*0401, and DRB1*1101 allotypes and found relatively conserved anchor residues at P1 and P4 and allele-specific anchor residues at P6. The common anchor residues at P1 were all aromatic residues. Because all three allotypes contain a G86 MHC residue near P1, this agrees with the above discussion of P1 preferences. The predominant residue observed at P4 was Met. All three allotypes have the A74 MHC residue variant making Met the most preferred residue at P4 according to the model parameters. Other MHC allotypes in the MultiRTA training set do not contain this variant so that the P4 residue preference observed in the study does not extend to other HLA-DR allotypes. This is reflected in the largest conserved interaction, as inferred by the largest magnitude parameter for the constant residue group, is Val at P4. At P6, the preference for Ala in DRB1*0101, Thr in DRB1*0401, and Arg in DRB1*1101 can be attributed to the allele-specific MHC residue variants W9, (H13, V11), and 11 S, respectively. These residue groups all appear in the other 11 MHC types included in the MultiRTA training set so that these P6 residue propensities are shared by other MHC allotypes in this larger context.

**Figure 1:**
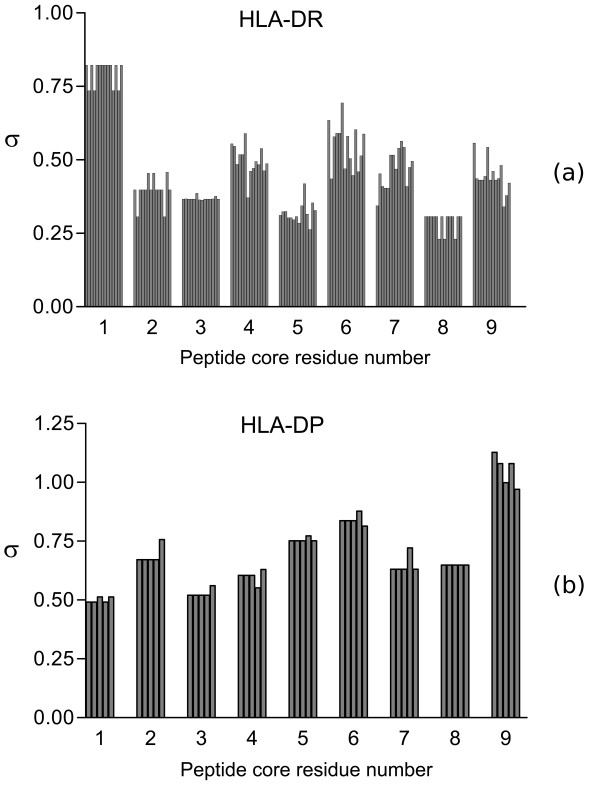
**Variation in the peptide core residue binding specificity as assessed by the standard deviation in MultiRTA predicted binding affinity over all 20 residue types**. Each bar represents the variation for one MHC allotype in the training data set.

We also examined the relative importance of each of the nine peptide core residues in binding specificity. This was done by calculating the standard deviation over all 20 residue types at core residue P*i *for each residue type *T *in the training sets, *σ_T, i _*defined in **Eq. 11**. These values are plotted for HLA-DR and HLA-DP in Figures 1(a) and 1(b), respectively. We also calculated the difference between the maximum and minimum binding affinity values and obtained qualitatively similar results (data not shown). For HLA-DR, it may be seen that P1 has the largest variation and so makes the largest contribution to binding specificity. This is consistent with the previous identification of P1 as a primary anchor residue for HLA-DR and with the discussion above. Peptide residues P4, P6, P7, and P9 also make large contributions to specificity relative to the remaining residues. Residue P4, P6, and P9 have been previously classified as secondary anchors on the basis of their contribution to binding specificity and the fact that they, along with P1 bind into four pockets in the MHC binding cleft. It is interesting that other peptide core residues, especially P7, also make significant contributions to peptide binding specificity. All nine peptide core side chains contact the MHC in some X-ray structure and so can potentially form energetically favorable interactions that stabilize peptide binding. Compared with peptide-class I MHC complexes, peptides bound to class II MHC assume an extended conformation low in the binding cleft rather than bulging outside of the binding cleft and so can form more extensive contacts with the MHC protein [46]. This also explains the general lack of well-defined anchor residues for class II MHC allotypes as compared with class I MHC. Thus, peptide core residues outside of the traditional anchor residues also make significant contributions to peptide binding to HLA-DR.

A similar analysis for HLA-DP reveals a different pattern of specificity from HLA-DR (see Figure 1(b)). In this case, P9 appears to contribute the most to binding specificity, with P2, P5, and P6 providing lesser but still significant specificity. Like HLA-DR, all peptide core positions provide non-negligible contributions to binding specificity. Taken as a whole, these trends disagree with two previous studies [37,50] that identified P1 and P6 as the primary anchor residues for the five HLA-DP allotypes in the training set. Although P6 is identified as an anchor residue, our analysis shows P1 to be one of the least important residues for specificity. The lower binding variation between the HLA-DP allotypes compared with the HLA-DR allotypes, previously described in Refs. [37,50], is likely due to the lower polymorphism of MHC residues interacting with the peptide.

### Conserved CLIP binding registers for HLA-DR and HLA-DP predicted by MultiRTA

In order to study an example of promiscuous binding across allotypes we analyzed MultiRTA predictions for the CLIP region of the p33 invariant chain (Ii), a naturally occurring promiscuous binder. All newly synthesized class II MHC proteins bind Ii in order to avoid prematurely loading peptides from the endoplasmic reticulum. The Ii peptide is later trimmed by proteases in the trans Golgi network to leave the bound CLIP fragment. In order to avoid inappropriate peptide binding and promote MHC complex assembly and transport [51], the CLIP peptide must form a stable complex with diverse class II MHC allotypes.

MultiRTA was used to make binding affinity predictions for the CLIP region (Ii, residues 81-104 (LPKPPKPVSKMRMATPLLMQALPM)) binding to all of the HLA-DR and HLA-DP training set allotypes. The identified primary core segments were MRMATPLLM (Ii 91-99) for all HLA-DR allotypes and RMATPLLMQ (Ii 92-100) for all HLA-DP allotypes examined. Ii 91-99 was also identified as a strongly binding secondary core segment for all HLA-DP allotypes, with contributions to the overall binding affinity only slightly lower (0.22 - 1.24 kcal/mol) than the primary core segment. Furthermore, the considerable variation in the binding affinities of CLIP to different HLA-DR allotypes is consistent with training data for longer CLIP region peptides and previous experimental binding assays [52]. In particular, HLA-DRB3*0101 is predicted to have markedly weak affinity for CLIP (IC_50_ = 6918 nM) compared with other HLA-DR allotypes, in agreement with Sette *et al*. 1995 [52].

The HLA-DR core segment is consistent with longer segments identified by many experiments on different HLA-DR types [52-55] and also agrees with an X-ray structure of the CLIP peptide bound to HLA-DR1*0301 [56]. One experimental study measured the concentration-dependent binding of a set of overlapping CLIP peptide segments to HLA-DPA1*0103/DPB1*0201 and concluded that Ii 91-99 is the core CLIP segment for this MHC type. Further experimental tests are needed to confirm whether or not Ii 92-100 constitutes an actual alternative binding register to HLA-DP. This is conceivable, as two alternative binding registers for CLIP have been experimentally identified for another class II allotype, HLA-DQ2 (HLA-DQA1*0501/DQB1*0201) [57].

## Conclusions

The MultiRTA model introduced in this paper generalizes our previously reported single-type RTA model to multiple related MHC allotypes. We fit both HLA-DR and HLA-DP models and found that the HLA-DR model achieved accuracy competitive with NetMHCIIpan, while using a much simpler and physically interpretable model of peptide-MHC binding. The HLA-DP multi-type model is the first of its kind, however limited variability between different allotypes combined with less available binding data yielded a model with considerably lower coverage than the HLA-DR model. In the future, expected additional experimental peptide binding data, particularly for distantly related MHC allotypes will expand the coverage of both models.

Other peptide-class II MHC binding prediction methods make use of additional peptide properties such as its length and the peptide flanking residues (PFRs) not used by MultiRTA. Including these properties in the MultiRTA model could possibly further improve its accuracy. For example, the NetMHCIIpan method uses the average BLOSUM scores over the peptide flanking residues (or PFRs, adjacent to the 9-mer core), lengths of the N- and C-terminal PFRs, and the peptide length. The inclusion of peptide length was previously shown to lead to potentially strong overfitting due to database-dependent length profiles [32] so that care is needed in interpreting any improvements in prediction performance with this property. The same study found that including information on PFRs further improved prediction performance. X-ray structures of peptide-MHC complexes show peptide-MHC residue interactions outside of the 9-mer peptide core, supporting the idea that PFRs can potentially make additional contributions to peptide binding affinity.

We were able to identify some of the most important determinants of both promiscuous and allele-specific peptide binding from the optimal MultiRTA parameters. While many deduced HLA-DR binding motifs agreed with previous studies, the relative importance of different HLA-DP core residue positions in determining binding specificity disagreed with two previous studies [37,50]. This difference may be due to the different method that we used to quantify the importance of each peptide core residue to the binding specificity. Both our analysis and those in the previous studies defined peptide residue positions with large variability in binding affinities as important for binding specificity, however the variability measures were different. In Caselli *et al*. 2002 [50] and Sidney *et al*. 2010 [37], variability was calculated from the binding affinities of single residue mutants of a reference peptide, whereas our analysis calculated the standard deviation in the MultiRTA parameters contributing to the predicted binding affinity at that position. If one assumes independent contributions of each peptide residue to the overall binding affinity, as is implicit in the MultiRTA model, then both approaches should yield the same qualitative result. Possible reasons for the discrepancy are unexpected shifts in the binding register for single mutants in the other analyses or inaccurate MultiRTA parameter values. Interestingly, the analysis of Sidney *et al*. 2010 [37] also found the peptide position two residues N-terminal to P1, which is outside of the 9-mer core, to be important for binding specificity, but less so than P1 and P6. This lends further support to that idea that accounting for PFRs in MultiRTA may improve its accuracy.

Overall, the analysis of important binding determinants showed that considerable sequence diversity is tolerated at the peptide core positions. This combined with uncertainty in the peptide binding register renders characterization of peptide binding specificity in terms of anchor residue preferences, which has proven useful for class I MHC, impractical for class II MHC. Thus more sophisticated descriptions of peptide binding preferences, such as MultiRTA, are needed for accurate predictions.

As mentioned above, the expected availability of additional experimental peptide binding data will improve the accuracy and coverage of the MultiRTA prediction models. Targeted analysis of peptide binding to MHC allotypes distantly related to allotypes represented in the current training data could expand coverage the most. As more binding data becomes available, an HLA-DQ model is also a possibility. However the large variability of the α chain, not present in HLA-DR and HLA-DP, will require a large quantity of experimental binding data for diverse allotypes in order to obtain a model with adequate coverage.

## Authors' contributions

AJB conceived of the study, collected the data sets, and analyzed the prediction results. HDM performed the numerical optimization to determine the model parameters. Both authors participated in drafting the manuscript and read and approved the final manuscript.

## Supplementary Material

Additional file 1 This table contains experimental peptide binding data for DRB1*1301, which was not included in the training set. All data were downloaded from IEDB [36]. Click here for file

Additional file 2 Tables S1 and S3 give the MHC residue group variants for each peptide core residue for HLA-DR and HLA-DP, respectively, while Tables S2 and S4 show all variations of peptide-contacting MHC residues appearing in the training set for HLA-DR and HLA-DP, respectively. Click here for file

Additional file 3 **This table contains a list of HLA-DR model parameters, β_ijl_, representing interactions between specific peptide and MHC residues**. They are divided by peptide core residue number and presented in decreasing order of magnitude. Click here for file

Additional file 4 **This table contains an ordered list of HLA-DP model interaction parameters in the same format as **Additional file 3. Click here for file
