# Supplementary material for: MultiRTA: A simple yet reliable method for predicting peptide binding affinities for multiple class II MHC allotypes
Source: BMC Bioinformatics. 2010 Sep 24;11:482. doi: 10.1186/1471-2105-11-482 (PMC2957400; doi:10.1186/1471-2105-11-482)
Supplement: Additional file 2 — Tables S1 and S3 give the MHC residue group variants for each peptide core residue for HLA-DR and HLA-DP, respectively, while Tables S2 and S4 show all variations of peptide-contacting MHC residues appearing in the training set for HLA-DR and HLA-DP, respectively. [file 1471-2105-11-482-S2.PDF]

Peptide core residue number	MHC contacting residue groups
P1	86G,86V
P2	77N,77T,78V,78Y,81H,81Y
P3	74A,74E,74L,74Q,74R,78V,78Y
P4	(11A,13C,26N,28I),(11D,28H),(11G,14K,74Q),(11L,26L),(11P,13R,71A),11R,11S,(11V,13H),13F,(13G,74L),13S,13Y,14E,26F,26Y,28D,28E,70D,70Q,(70R,74E),71E,71K,71R,74A,74R,78V,78Y
P5	(11A,13C,28I),(11D,28H),(11G,30L,74Q),(11L,30C),(11P,13R,71A),11R,11S,(11V,13H),13F,(13G,74L),13S,13Y,28D,28E,30D,30G,30Y,70D,70Q,(70R,74E),71E,71K,71R,74A,74R
P6	9E,(9K,30G),(9Q,30D),9W,(11A,13C),11D,(11G,30L),(11L,30C),(11P,13R,71A),11R,11S,(11V,13H),13F,13G,13S,13Y,30Y,71E,71K,71R
P7	28D,28E,28H,28I,30C,30D,30G,30L,30Y,47F,47Y,67F,67I,67L,70D,70Q,70R,71A,71E,71K,71R
P8	60S,60Y
P9	9E,(9K,30G),(9Q,30D,37D),9W,30C,30L,30Y,37F,37N,37S,37Y,38A,38L,38V,57D,57S,(57V,60S),60Y

Table S1: MHC contacting residue groups for each of the 9 peptide core residue positions for the 14 HLA-DR allotypes in the training set.

MHC residue number	Residue types
9	QKEW
11	ADGLPSRV
13	CGFHSRY
14	KE
26	YNLF
28	IHED
30	YCDGL
37	YSFDN
38	ALV
47	YF
57	SDV
60	YS
67	ILF
70	QRD
71	AKRE
74	AQREL
77	TN
78	YV
81	YH
86	GV

Table S2: Residue types appearing at the specified residue number in the 14 HLA-DR MHC allotypes included in the training set.

Peptide core residue number	MHC contacting residue groups
P1	(86G,89M),(86D,89V)
P2	78M,78V
P3	78M,78V
P4	71E,71K,71V,78M
P5	71E,71K
P6	9F,9Y,71E,71K
P7	71E,71K
P8	None
P9	9F,37F,38V,57D,(9Y,37Y),(37L,57E),(38A,57A)

Table S3: MHC contacting residue groups for each of the 9 peptide core residue positions for the five HLA-DP allotypes in the training set.

MHC residue number	Residue types
9	YHF
11	LG
15	CG
37	YLF
38	AV
57	AED
67	IL
71	KRE
78	IMV
86	DGV
89	MV

Table S4: Residue types appearing at the specified residue number in the five HLA-DP MHC allotypes included in the training set.
